# Insights into the Interactions between Maleimide Derivates and GSK3β Combining Molecular Docking and QSAR

**DOI:** 10.1371/journal.pone.0102212

**Published:** 2014-07-10

**Authors:** Luisa Quesada-Romero, Karel Mena-Ulecia, William Tiznado, Julio Caballero

**Affiliations:** 1 Centro de Bioinformática y Simulación Molecular, Facultad de Ingeniería, Universidad de Talca, Talca, Chile; 2 Departamento de Ciencias Químicas, Facultad de Ciencias Exactas, Universidad Andres Bello, Santiago de Chile, Chile; Xuzhou Medical college, China

## Abstract

Many protein kinase (PK) inhibitors have been reported in recent years, but only a few have been approved for clinical use. The understanding of the available molecular information using computational tools is an alternative to contribute to this process. With this in mind, we studied the binding modes of 77 maleimide derivates inside the PK glycogen synthase kinase 3 beta (GSK3β) using docking experiments. We found that the orientations that these compounds adopt inside GSK3β binding site prioritize the formation of hydrogen bond (HB) interactions between the maleimide group and the residues at the hinge region (residues Val135 and Asp133), and adopt propeller-like conformations (where the maleimide is the propeller axis and the heterocyclic substituents are two slanted blades). In addition, quantitative structure–activity relationship (QSAR) models using CoMSIA methodology were constructed to explain the trend of the GSK3β inhibitory activities for the studied compounds. We found a model to explain the structure–activity relationship of non-cyclic maleimide (NCM) derivatives (54 compounds). The best CoMSIA model (training set included 44 compounds) included steric, hydrophobic, and HB donor fields and had a good *Q^2^* value of 0.539. It also predicted adequately the most active compounds contained in the test set. Furthermore, the analysis of the plots of the steric CoMSIA field describes the elements involved in the differential potency of the inhibitors that can be considered for the selection of suitable inhibitors.

## Introduction

Glycogen synthase kinase-3 (GSK3) is a widely expressed and multifunctional serine/threonine protein kinase involved in a large number of cellular processes and diseases. GSK3 is regulated by several mechanisms including phosphorylation [1], intracellular localization [2], and protein complex formation [3]. On the other hand, GSK3 regulates many cellular processes such as cellular architecture and motility [4], and contributes to cell death and cell survival [5], [6]. In the last decades, GSK3 has been extensively investigated because its dysregulation is associated to several diseases including Alzheimer’s disease [7], diabetes [7], [8], cancer [3], muscle hypertrophy [9], etc.

GSK3 is encoded by two isoforms in mammals named GSK3α and GSK3β [10]. Both isoforms have almost identical catalytic domains, they are activated by tyrosine phosphorylation (Tyr279/216 in GSK3α/GSK3β) and are inhibited by phosphorylation in Ser21 in GSK3α and Ser9 and Thr390 in GSK3β) [11]. Different roles in diseases have been identified for each isoform: for instance, GSK3β is overexpressed in many types of cancer including ovarian cancer [12], pancreatic cancer [13], colon cancer [14], etc; meanwhile, there are few reports on the role of GSK3α in cancer [15].

GSK3β has been proposed as a target for therapy in order to combat several diseases. Many small organic chemical compounds have been developed as ATP competitive GSK3β inhibitors [16]. Among them, a series of macrocyclic and non-cyclic maleimide derivatives (MCMs and NCMs) was reported, giving some candidates with high potency and selectivity [17]–[21]. In this work, we modeled the structure of the complexes between GSK3β and these compounds using docking. Active conformations are proposed and the interactions that contribute to form the complexes are described. We also develop quantitative structure–activity relationship (QSAR) models using CoMSIA method. The combined docking-CoMSIA protocol is used to provide information about the structural features of potent inhibitors. With this information, we speculated on the possible causes of differential biological activities.

## Materials and Methods

### Data set

**Figure 1:**
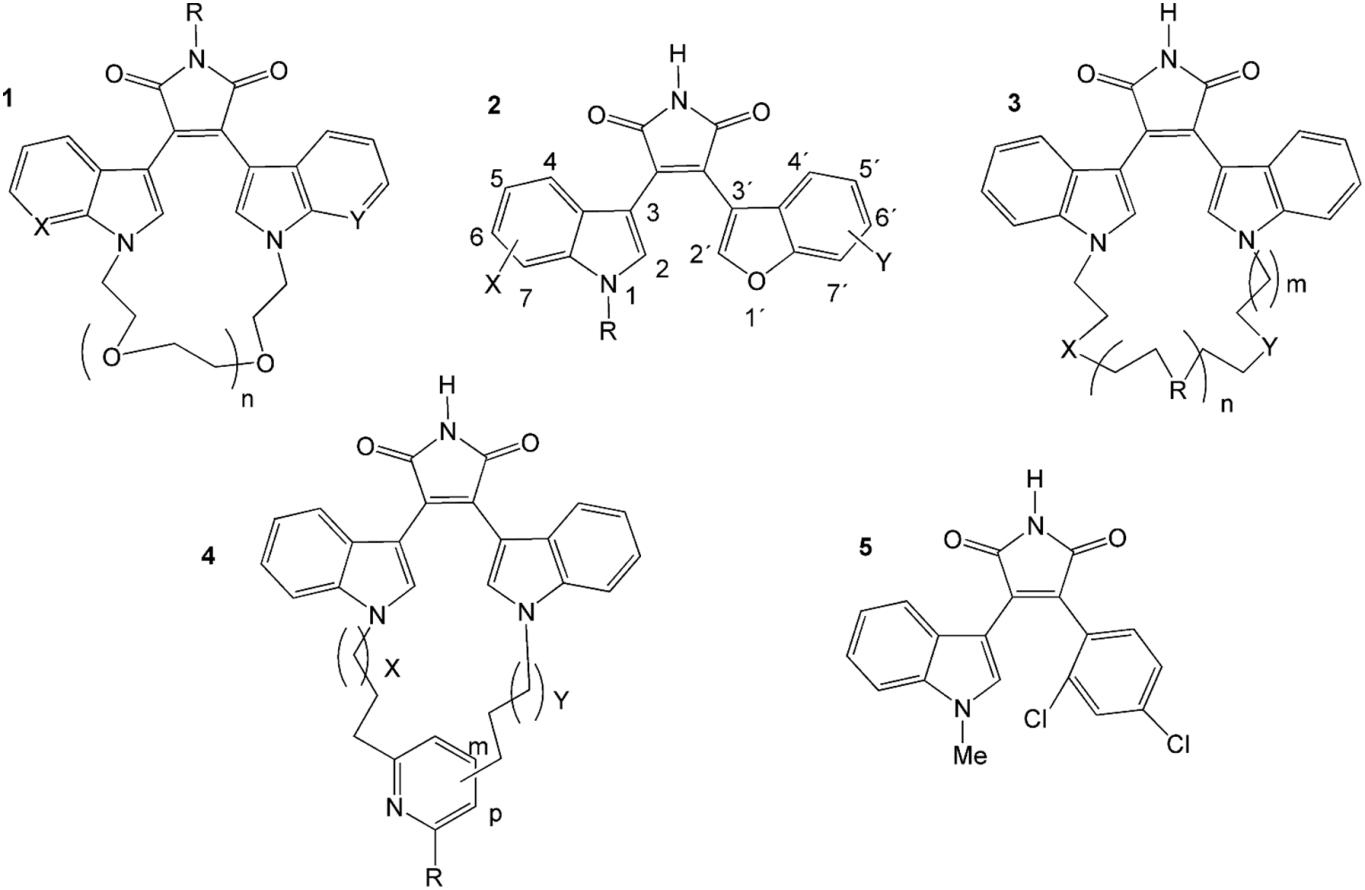
Structures of MCMs (1, 3, and 4) and NCMs (2 and 5).

The structures and GSK3β inhibitory activities of 23 MCMs and 54 NCMs were collected from the literature [17]–[20]. The tridimensional (3D) structures were sketched using Maestro’s molecular editor (Maestro 9.0, Schrödinger LLC). Activities were collected and transformed into log(10^3^/IC_50_) values, where IC_50_ values represent the compound µM concentrations that inhibit the GSK3β activity by 50%. The compounds under study and their inhibitory biological activities are summarized in Figure 1 and Table 1.

**Table 1:** Experimental and predicted GSK3β inhibitory activities (log(10^3^/IC_50_)) of MCMs and NCMs using model CoMSIA-NCM-SHD.

Compounds^a^	R	X	Y	n	m	log(10^3^/IC_50_)	
						Exp.	Pred.^b^
**1a**	H	C	C	1	-	3.866	-
**1b**	H	C	C	2	-	4.657	-
**1c**	H	C	C	4	-	4.292	-
**1d**	H	C	N	1	-	3.860	-
**1e**	H	C	N	2	-	4.769	-
**1f**	H	N	N	1	-	3.207	-
**1g**	H	N	N	2	-	4.468	-
**1h**	H	N	N	3	-	4.318	-
**2a**	CH_3_	H	H	-	-	4.455	4.203
**2b**	CH_3_	5-Br	H	-	-	5.154	4.761
**2c**	CH_3_	6-OH	H	-	-	4.823	4.741
**2d**	CH_3_	6-OH	5′-F	-	-	4.853	4.810
**2e** c	CH_3_	5-C≡CH	H	-	-	5.017	3.995
**2f**	H	H	5′-F	-	-	3.173	3.521
**2g** c	CH_3_	H	5′-Br	-	-	3.259	5.481
**2h**	CH_3_	H	7-OCH_3_	-	-	3.744	4.277
**2i**	H	5-F	H	-	-	3.444	3.332
**2j** c	CH_3_	5-F	H	-	-	4.585	5.077
**2k**	CH_3_	5-Cl	5′-F	-	-	4.377	4.520
**2l**	CH_3_	5-OCH_3_	H	-	-	3.903	3.682
**2m**	H	5-OBn	H	-	-	2.783	2.900
**2n**	CH_3_	5-OBn	H	-	-	3.301	3.060
**2o**	(CH_2_)_3_OH	5-OBn	H	-	-	3.657	3.703
**2p**	CH_3_	6-OBn	H	-	-	3.045	3.384
**2q** c	CH_3_	6-OBn	5′-F	-	-	3.796	2.948
**2r**	CH_3_	7-OBn	H	-	-	3.657	3.597
**2s**	CH_3_	5-cyclopropane	H	-	-	3.628	3.771
**2t**	CH_3_	5-OH	H	-	-	3.161	3.238
**2u** c	CH_3_	7-OH	H	-	-	4.260	5.977
**2v**	CH_3_	5-F	6′-CH_2_OH	-	-	6.455	6.596
**2w**	CH_3_	5-F	6′-CH_2_OCH_3_	-	-	4.623	4.491
**2x**	CH_3_	5-F	6′-OH	-	-	5.455	5.715
**2y**	CH_3_	5-F, 6-I	7′-OCH_3_	-	-	3.607	3.472
**2z**	CH_3_	5-F, 6-Cl	H	-	-	3.735	3.904
**2aa**	CH_3_	5-F, 6-Cl	6′-CH_2_OH	-	-	6.022	6.124
**2ab**	CH_3_	5-F, 6-Cl	6′-0CH_3_	-	-	3.060	3.326
**2ac**	CH_3_	5-F, 6-Cl	6′-cyclopropylmethoxy	-	-	2.889	2.599
**2ad**	CH_3_	5-F, 6-Cl	6′-cyclobutylmethoxy	-	-	2.388	2.394
**2ae** c	CH_3_	5-F, 6-Cl	7′-OCH_3_	-	-	3.585	4.411
**2af**	CH_3_	5-F, 6-p-Cl-Ph	7′-OCH_3_	-	-	2.145	2.143
**2ag**	CH_3_	5-Br	7′-OCH_3_	-	-	5.124	5.262
**2ah**	CH_3_	5-Br	6′-CH_2_OH	-	-	6.292	6.333
**2ai**	CH_3_	5-Br	6′-prop-2-ynyloxy	-	-	4.596	4.494
**2aj**	CH_3_	5-Br	6′-allyloxy	-	-	4.316	4.372
**2ak**	CH_3_	5-Br	6′-O-(p-CH_3_O)-Bn	-	-	3.474	3.430
**2al**	CH_3_	5,7-dibromo	7′-OCH_3_	-	-	4.052	4.034
**2am** c	CH_3_	5-I	H	-	-	4.462	4.531
**2an** c	CH_3_	5-I	5′-F	-	-	3.744	4.670
**2ao**	CH_3_	5-CN	6′-CH_2_OH	-	-	4.879	5.009
**2ap**	CH_3_	5-cyclopropylethynyl	5′-F	-	-	4.793	4.807
**2aq**	CH_3_	5,6-methylenedioxy	5′-F	-	-	3.148	3.511
**2ar**	CH_3_	5-OCH_3_, 6-Cl	H	-	-	3.356	3.077
**2as**	CH_3_	5-OCH_3_, 6-I	H	-	-	3.651	3.611
**2at** c	CH_3_	6-CF_3_	7′-OCH_3_	-	-	3.080	3.888
**2au**	CH_3_	7-CH_2_OH	H	-	-	5.267	4.997
**2av**	CH_3_	7-CH_2_OH	6′-CH_2_OH	-	-	5.292	5.441
**2aw**	CH_3_	7-CH_2_OMe	H	-	-	6.638	6.315
**2ax** c	CH_3_	7-CH_2_OMe	6′-CH_2_OH	-	-	6.136	7.371
**2ay**	CH_3_	7-CH_2_CH_2_COOEt	H	-	-	4.991	4.777
**2az**	CH_3_	7-CH_2_CH_2_COOH	H	-	-	5.920	5.748
**2ba**	CH_3_	*1*H-benzo[g]	5′,6′-difluoro	-	-	3.503	3.513
**3a**	NEt	O	O	1	1	4.602	-
**3b**	NMe	O	O	1	1	4.149	-
**3c**	NMe	NMe	O	1	1	4.481	-
**3d**	NMe	NMe	NMe	1	1	4.367	-
**3e**	O	O	NMe	1	2	5.398	-
**3f**	O	O	NEt	1	1	5.398	-
**3g**	None	NMe	NMe	0	1	3.959	-
**3h**	O	NMe	NMe	1	1	5.222	-
**3i**	O	NMe	NMe	2	1	5.097	-
**4a**	Me_2_N	1	1	-	*meta*	5.155	-
**4b**	(CH_2_)_4_N	1	1	-	*meta*	4.959	-
**4c**	Me_2_N	1	1	-	*para*	4.432	-
**4d**	(CH_2_)_4_N	1	1	-	*para*	4.523	-
**4e**	Me_2_N	1	2	-	*para*	5.523	-
**4f**	(CH_2_)_4_N	2	2	-	*para*	4.620	-
**5**	-	-	-	-	-	4.301	4.155

a Compounds **1** from reference [17], compounds **2a–2u** from reference [19], compounds **2v**–**2ba** and **5** from reference [21], compounds **3** from reference [20], and compounds **4** from reference [18].

b Predictions using model CoMSIA-NCM-SHD.

c Compounds predicted in the test set.

### Docking

Generally, docking algorithms reproduce the bound form of ligands inside the active site of proteins [22]–[30]. In our current work, docking was performed using Glide [31]. Protein coordinates were extracted from the X-ray crystal structure of the GSK3β-inhibitor complex with the code 2OW3 in Protein Data Bank [18]. A grid box of 30Å×30Å×30Å was centered on the center of mass of the inhibitor in this crystal structure covering the ATP-binding site of the enzyme. The module LigPrep 2.5 [32] was used to assign ionization states, stereochemistries, and ring conformations of the sketched ligands. Docking parameters were used as in previous works [29], and Glide standard (SP) and extra-precision (XP) modes were explored during the search. The better docking poses for each ligand were analyzed by examining their relative total energy scores. Among docking poses, the more energetically favorable conformation was selected by considering the total energy value.

### QSAR modeling

CoMSIA was applied to selected training set, and then external set was predicted. Firstly, CoMSIA was performed on the whole dataset including 64 compounds in the training set and 13 compounds in the external set, but we did not find a proper result. Therefore, we constructed separated CoMSIA models describing the structure-activity relationships of MCMs and NCMs. MCMs (23 compounds) were modeled without splitting of the dataset, and NCMs (54 compounds) were modeled including 44 compounds in the training set and 10 compounds in the external set.

CoMSIA was performed using the Sybyl 7.3 software of Tripos [33]. Field descriptors were calculated on the 3D conformations obtained by the docking approach; this guaranteed that all compounds were aligned in the GSK3β active site. The maleimide derivatives were placed in a rectangular grid extended beyond 4 Å in each direction from the coordinates of each molecule. The interaction energies between a probe atom (an sp^3^-hybridized carbon atom with +1 charge) and all compounds were computed at the surrounding points, using a volume-dependent lattice with 2.0-Å grid spacing. Similarity is expressed in terms of steric occupancy, electrostatic interactions, local hydrophobicity, and hydrogen bond (HB) donor and acceptor properties, using a 0.3 attenuation factor. The number of components in PLS was optimized using the *Q* ^2^ value, obtained from the leave-one-out (LOO) crossvalidation procedure, with the SAMPLS [34] sampling method.

## Results and Discussion

### Docking results

**Figure 2:**
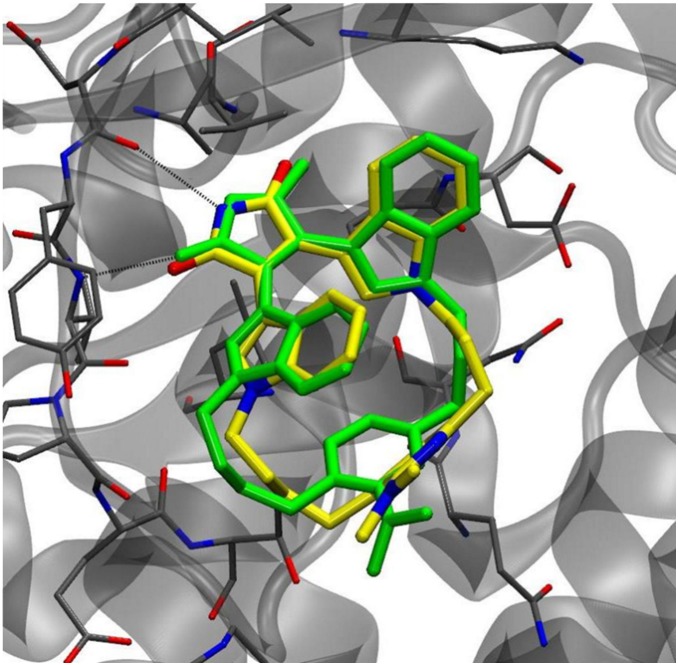
Conformational comparison of compound 4e from the crystal structures in GSK3β–inhibitor complex (inhibitor in yellow in online version, light gray in printed version) and from docking results (inhibitor in green in online version, dark gray in printed version).

We tested the ability of the docking method to reproduce the pose of compound **4e** close to that found in an X-ray complex reported in reference [18]. As it can be observed in Figure 2, the docked structure closely corresponds to the inhibitor in the X-ray structure 2OW3 (root mean square deviation, RMSD, of all heavy atoms: 0.910 Å). Therefore, we can state that Glide software found a correct binding mode of the studied ligand.

**Figure 3:**
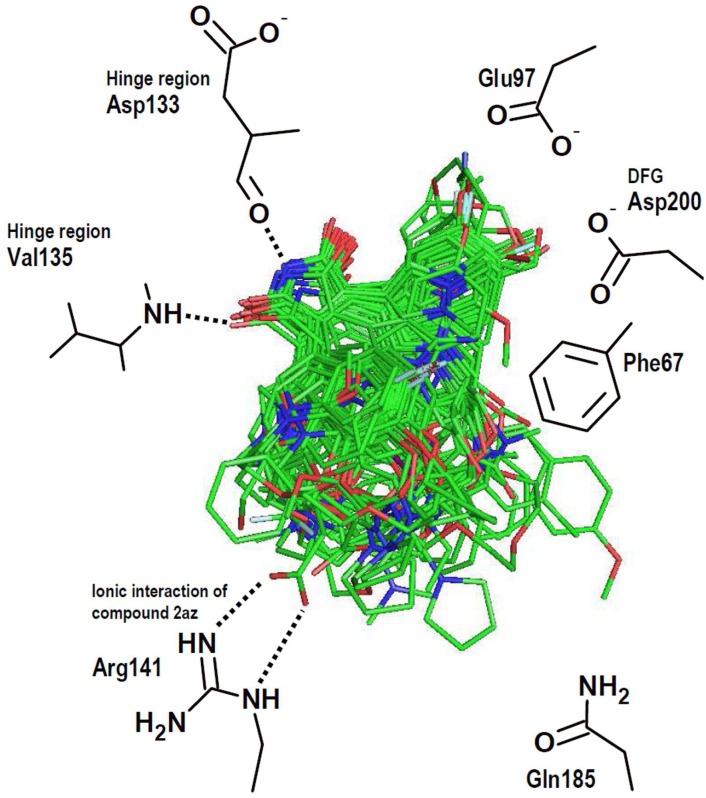
Predicted binding conformations of all the investigated maleimide derivatives and their interactions with the residues in the GSK3β active site.

The alignment of the docked structures inside the GSK3β binding site for the remaining studied compounds is shown in Figure 3. The docking protocol was successful since all the ligands were docked in the expected orientations. In general, our docking results show that all the maleimide derivates adopt the same mode of binding, characterized by interactions of the maleimide carbonyl and NH groups with residues in the GSK3β hinge region (Figure 3): one of the carbonyl groups forms an HB interaction with the backbone NH of the residue Val135, and the maleimide NH group forms an HB interaction with the backbone carbonyl group of Asp133. In general, the studied compounds contain heterocyclic substituents (benzofuran-3-yl or indol-3-yl) at positions 3 and 4 of maleimide. One of the heterocyclic substituents is located close to the residues Asp200 (DFG motif), Lys85 (catalytic lysine), and Cys199; the other is oriented towards the surface of the pocket (in the region between the residues Ile62 and Leu188). All the MCMs (series **1**, **3**, and **4**) contain two indol-3-yl (or other nitrogen containing heterocycles) substituents. The groups are oriented so as to accommodate the macrocyclic linker in the region between the residues Phe67, Thr138, and Gln185. In this zone the macrocyclic linker is largely solvent-exposed.

Compounds of series **1** contain a polyoxygenated macrocyclic linker and have log(10^3^/IC_50_) between 3.2 and 4.8; while compounds of series **3** contain multiple heteroatoms on the macrocyclic linker, and have log(10^3^/IC_50_) between 3.9 and 5.4. The orientations of the poses obtained for these series suggest that HB donor groups at the macrocyclic linkers could interact with the side chain NH_2_ group of the residue Asn186. On the other hand, compounds of series **4** are macrocyclic pyridinophanes and have log(10^3^/IC_50_) between 4.4 and 5.6. The groups that differentiate the activity in this series are exposed to the solvent close to the residues Gln185 and Thr138. The obtained docking poses do not reveal the source of the differential activity among compounds of the series **4**, since the pyridinophane groups are not involved in important contacts with the protein.

**Figure 4:**
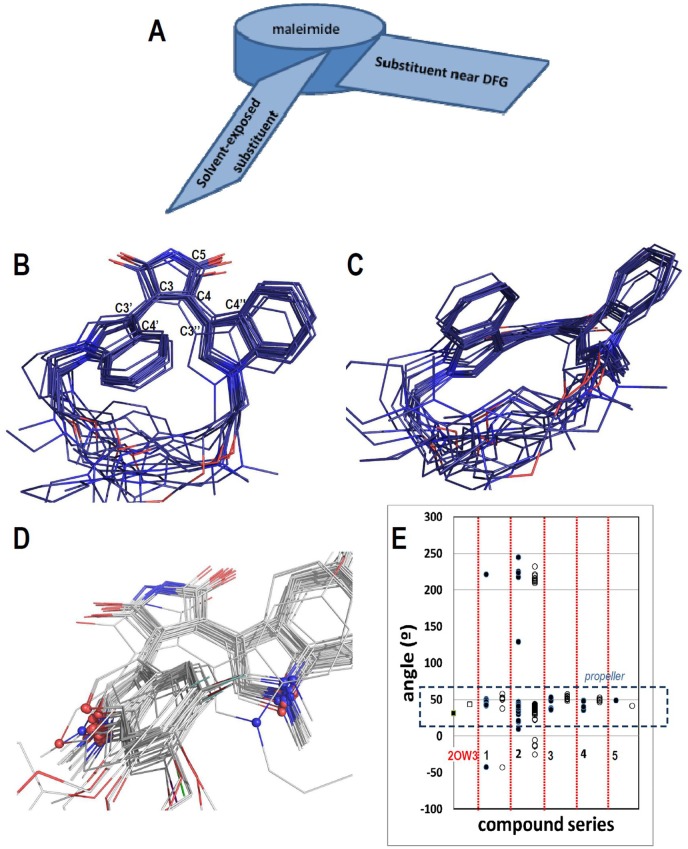
Analysis of the propeller conformations of the docked maleimides. (A) Scheme of the propeller conformations. (B, C) Two views of the propeller conformations of MCMs. (D) Solvent-exposed substituents (at the left) and substituents near the DFG Asp200 (at the right) of NCMs showing the presence of indol-3-yl and benzofuran-3-yl groups at each position. (E) Dihedral angles Angle1 C4’-C3′-C3-C4 (close circles to the left) and Angle2 C4″-C3″-C4-C5 (open circles to the right) for each compound series (consider labels of the atoms as represented in Figure 3A). The same dihedral angles are represented for the crystallographic structure of **4e** (close and open squares, PDB code: 2OW3). The dashed lines box encloses the propeller conformations.

A remarkable feature of the obtained docking poses is that 3, 4 disubstituted maleimides form a propeller-like conformation inside the GSK3β binding site: the propeller axis (the maleimide) with two slanted blades (the heterocyclic substituents), as represented in the Figure 4A. Figures 4B and 4C show two views of the propeller conformations for MCMs; where the indol-3-yl groups at the left are solvent-exposed and the indol-3-yl groups at the right are near the DFG motif (considering the positions of the inhibitors inside the GSK3β binding site). The figure 4D shows the propeller conformations for NCMs. In general, the benzofuran-3-yl groups are solvent-exposed and indol-3-yl groups are near the DFG motif, but several compounds exchange these common orientations, which is reasonable considering the symmetry of the NCMs. The inclination of the blades (heterocyclic substituents) was analyzed for each series by measuring dihedral angles C4′-C3′-C3-C4 (Angle1) and C4″-C3″-C4-C5 (Angle2) defined in Figure 4B. The results are plotted in Figure 4E, where it is possible to see that the inclinations that lead to propeller conformations are characterized by Angle1 and Angle2 values between 30 and 56°. All the compounds from series **3** and **4** have perfect propeller conformations (Angle1 = 44.8°±7.1 and 42.8°±6.2; Angle2 = 52.5°±2.8 and 49.4°±2.1, for series **3** and **4**, respectively). The majority of compounds from series **1** also have propeller conformations (Angle1 = 46.0°±3.8; Angle2 = 51.8°±0.9). In general, the analysis of the docking poses for MCMs (compounds from series **1**, **3**, and **4**) shows that the heterocyclic substituents near the DFG motif lean at higher angle values with respect to solvent-exposed ones. We consider that this is an effect of the rigid macrocyclic linkers. It is noteworthy that the known co-crystallized structure of compound **4e** in GSK3β (pdb: 3OW3) has slightly lesser values for Angle1 (31.2°) and Angle2 (43.7°), but Angle2 is higher than Angle1 in accordance with docking results. The majority of compounds from series **2** also have propeller conformations, but Angle1 and Angle2 have similar values for these compounds (Angle1 = 40.4°±2.5; Angle2 = 39.6°±3.3). Finally, Angle1 and Angle2 are 48.8 and 41.2 respectively for the docking pose of compound **5**. According to this analysis, NCMs (compounds of series **2** and compound **5**) also have propeller conformations with less inclined blades inside GSK3β.

In some cases, the presence or absence of propeller conformations are due to the formation of HBs with polar groups in the active site. For instance, compounds that contain CH_2_OH substituent at position 6 of the indol-3-yl near the DFG motif, such as **2ah**, **2au**, and **2av**, form the propeller conformation with an additional HB between the CH_2_OH and the catalytic lysine (Lys85) or DFG aspartate (Asp200). On the other hand, compounds that contain CH_2_OH substituent at position 6 of the solvent-exposed indol-3-yl, such as **2v** and **2aa**, form the propeller conformation with an additional HB between the CH_2_OH and the backbone carbonyl of the residue Ile62. It is noteworthy that the majority of compounds with CH_2_OH substituent at position 6 of the indol-3-yl have log(10^3^/IC_50_) above 6. A striking case is the compound **2az** that contains a carboxylate group and a high log(10^3^/IC_50_) value of 5.920. This compound has the propeller conformation and establishes an interesting ionic interaction between the carboxylate group and the residue Arg141 of GSK3β (it is indicated in the Figure 3). During the analysis of docking poses, we identified that compounds that have bulky substituents, such as **2m**, **2n**, **2q**, **2s**, **2ap**, and **2ay**, do not have a propeller conformation due to steric problems. In general, these compounds are less active.

### QSAR models

We also constructed CoMSIA models to identify the structural features of the maleimide derivatives that affect their inhibitory activity against GSK3β. The models were developed by using the docking aligned conformations, allowing the CoMSIA contours display into the GSK3β active site. Models were derived from different combinations of up to five fields. The best model was selected by the analysis of the statistical quality of the internal LOO cross-validation for each model, taking into account *Q^2^* values. We developed models for describing the whole dataset (77 compounds), the MCMs (23 compounds), and NCMs (54 compounds), after performing the splittings mentioned above in the Materials and methods section.

**Figure 5:**
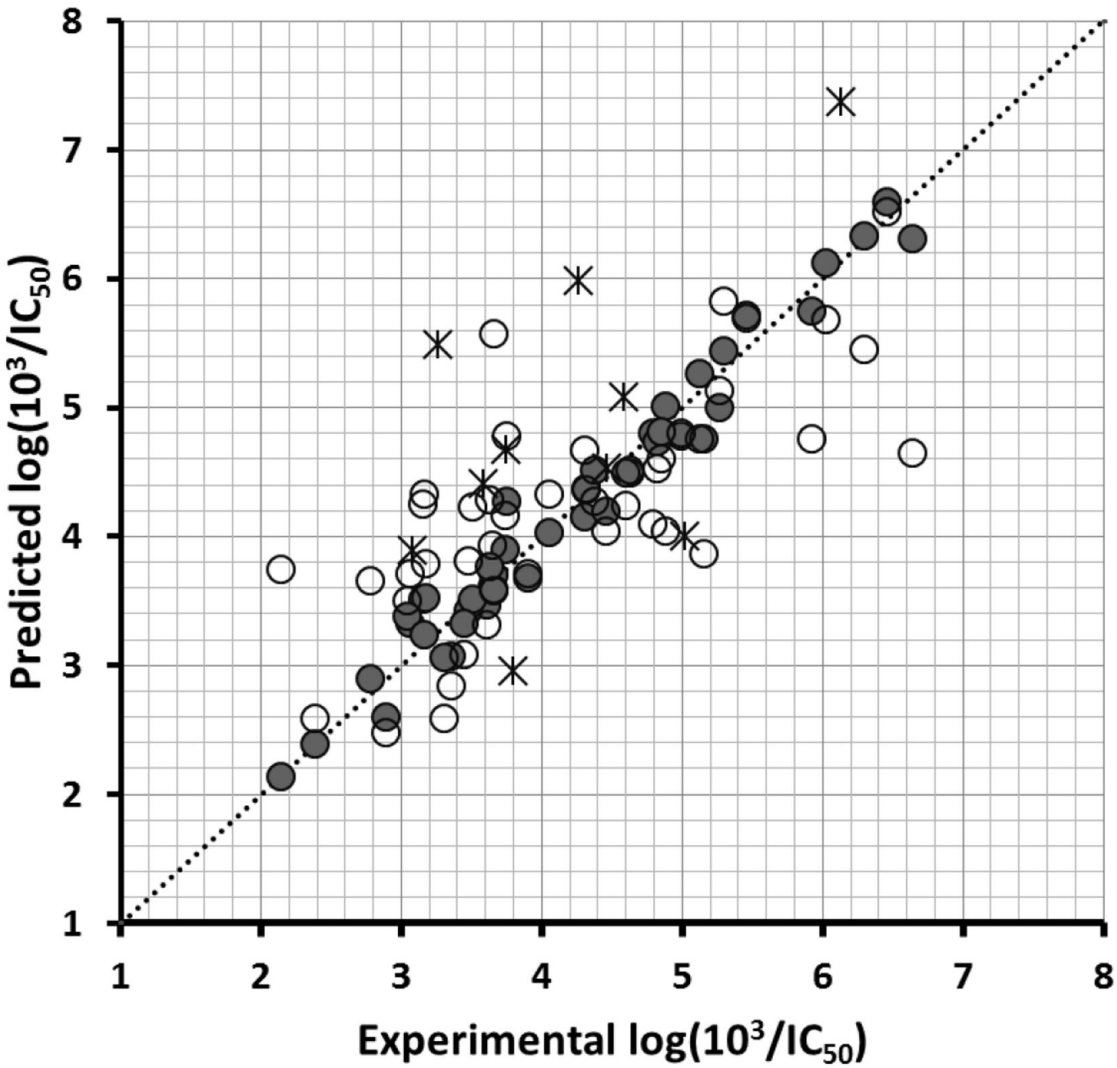
Scatter plot of the experimental activities versus predicted activities for model CoMSIA-NCM-SHD: (•) training-set predictions, (○) LOO cross-validated predictions, (×) test-set predictions.

The results of the search are included in Table 2. We could not find predictive models for describing the structure-activity relationship of the whole dataset and the subset of MCMs. On the other hand, we found the model CoMSIA-NCM-SHD (*Q^2^* = 0.539) for describing the differential GSK3β inhibitory activities of NCMs. This model uses five components and combines steric, hydrophobic, and HB donor fields with contributions of 17.1%, 56.2%, and 26.7% respectively. In addition, it explains 92.1 of the variance and has a low standard deviation (S = 0.329) and a high Fischer ratio (F = 89.13). The predictions of log(10^3^/IC_50_) values for the 44 NCMs from the training set using model CoMSIA-NCM-SHD are shown in Table 1. The correlations between the calculated and experimental log(10^3^/IC_50_) values (from training and LOO cross-validation) are shown in Figure 5. According to these plots, the model properly discriminates between the most and less active compounds. We also predicted the GSK3β inhibitory activities of the test set compounds using model CoMSIA-NCM-SHD. The obtained prediction values are given in Table 1, and correlation between the calculated and experimental values is represented in Figure 5. This analysis reveals that the proposed model identified the most active compounds in the test set, but certainly not a good correlation was obtained when *R^2^* of the test set was analyzed. Therefore, the predictive evaluation of this model using rigorous external validation testing (calculation of *R^2^_m_* [35]) lead to unfavorable results.

**Table 2:** CoMSIA results using the best field combinations.^a^

Models	NC	R^2^	S	F	Q^2^	S_cv_	Fraction				
							S	E	H	D	A
CoMSIA-SHD	5	0.843	0.410	62.17	0.304	0.863	0.208		0.549	0.243	
CoMSIA-SEHD	5	0.901	0.325	106.02	0.251	0.895	0.112	0.380	0.338	0.170	
CoMSIA-SH	5	0.759	0.508	36.52	0.236	0.904	0.291		0.709		
CoMSIA-MCM-SA	1	0.492	0.416	20.36	0.217	0.516	0.171				0.829
CoMSIA-MCM-A	1	0.464	0.427	18.18	0.206	0.520					1
CoMSIA-MCM-SDA	1	0.547	0.393	25.37	0.184	0.527	0.142			0.168	0.690
**CoMSIA-NCM-SHD**	**5**	**0.921**	**0.329**	**89.13**	**0.539**	**0.802**	**0.171**		**0.562**	**0.267**	
CoMSIA-NCM-HD	5	0.908	0.355	75.39	0.520	0.813			0.697	0.303	
CoMSIA-NCM-EHD	5	0.945	0.275	131.22	0.459	0.863		0.380	0.418	0.202	

a NC is the number of components from the PLS analysis; *R* ^2^ is the square of the correlation coefficient; *S* is the standard deviation of the regression; *F* is the Fischer ratio; and *Q* ^2^ and *S*_cv_ are the correlation coefficient and standard deviation, respectively, of the leave-one-out (LOO) cross-validation.

**Figure 6:**
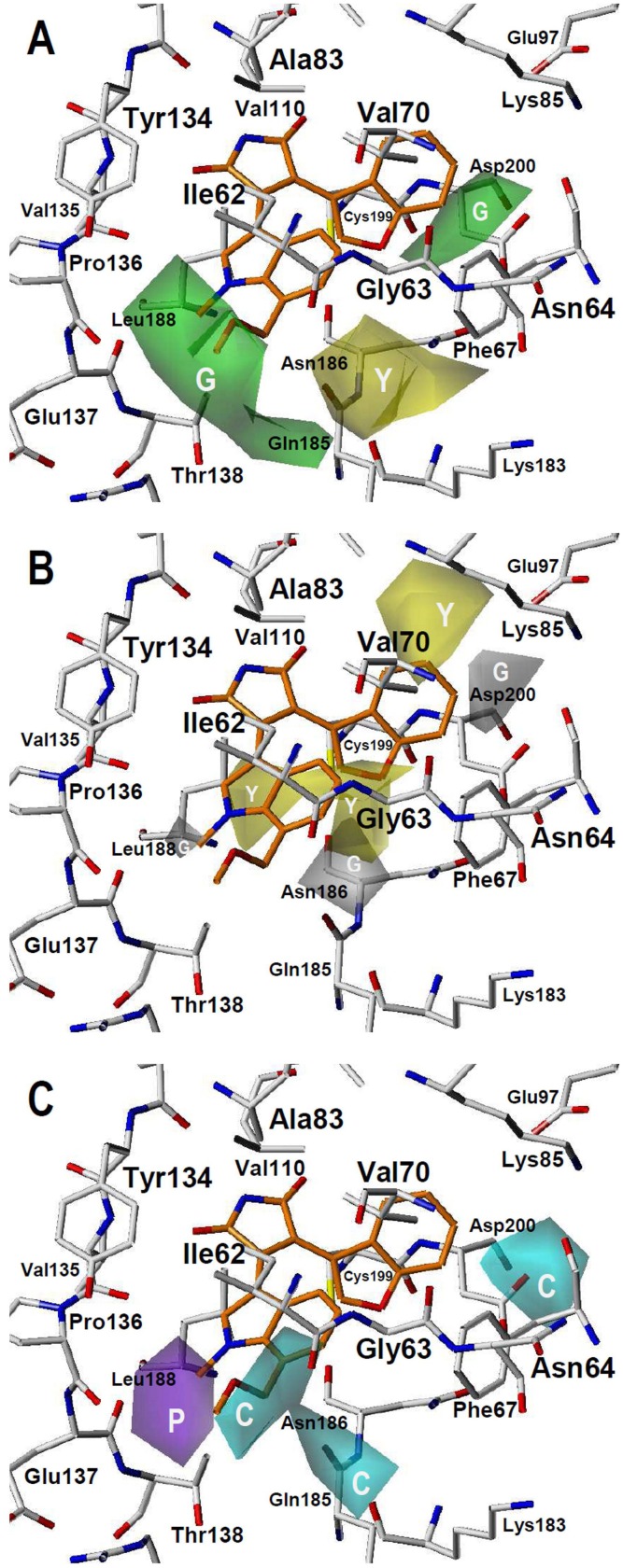
CoMSIA contour maps for GSK3β inhibitors deriving from model CoMSIA-NCM-SHD. The amino acid residues located close to the binding pocket of GSK3β are represented for comparing their position with the position of isopleths derived from the model. Compound **2aw** is shown inside the fields. (A) Steric field: green isopleths indicate regions where bulky groups favor the activity, and yellow isopleths indicate regions where bulky groups disfavor the activity. (B) Hydrophobic field: yellow isopleths indicate regions where hydrophobic groups favor the activity, and gray isopleths indicate regions where hydrophilic groups favor the activity. (C) HB donor field: cyan isopleths indicate regions where HB donors favor the activity, and purple isopleths indicate regions where HB donors disfavor the activity.

The contour plots of the CoMSIA steric, hydrophobic, and HB donor fields are presented in Figure 6 for the best model CoMSIA-NCM-SHD. The highly active compound **2aw** is displayed in the maps to aid in visualization, and the superposition of CoMSIA contour plots on active-site residues is also shown. The colored isopleths in the map represent the 3D locations where the structural properties changes are related to the changes in biological potency. Green and yellow isopleths in Figure 6A indicate regions where bulky groups increase and decrease the inhibitory activity, respectively. A large region of green contour near the solvent-exposed indol-3-yl moiety of compound **2aw** suggests that there is a favorable steric region near the residues Leu188, Thr138, and Gln185. In fact, compound **2aw** has methyl and methoxymethyl substituents at positions 1 and 7 respectively of the indol-3-yl moiety that occupy this region. Other green isopleth near the residues Asp200 and Phe67 indicates that bulky groups at positions 6 and 7 on the indol-3-yl or benzofuran-3-yl in the neighborhood of the DFG motif increase the inhibitory activity. This is the case of active compounds that contain hydroxymethyl group; for instance, compound **2av** (log(10^3^/IC_50_) = 5.292) contains this group at position 7 of the indol-3-yl substituent, and compound **2ah** (log(10^3^/IC_50_) = 6.292) contains this group at position 6 of the benzofuran-3-yl substituent. Finally, the yellow isopleth near the residue Asn186 suggests that large groups are not desired in this zone. It must be remembered that bulky groups that impair the formation of the propeller conformation occupy this region; for instance, the less active compound **2ad** (log(10^3^/IC_50_) = 2.388) contains a cyclobutyl in this region.

Yellow isopleths in Figure 6B indicate regions where hydrophobic groups are favorable for activity or hydrophilic groups diminish the activity; meanwhile, gray isopleths represent areas where hydrophobic groups are not favorable for activity or hydrophilic groups increase the activity. According to the analysis of the yellow isopleths, hydrophobic groups are tolerated in the region in front of the positions 2 and 3 of the maleimide. Several active compounds contain a methyl group at position 1 of the indol-3-yl near the DFG motif in this region. There is other yellow isopleth near the catalytic lysine (Lys85) that is occupied by halogen substituents at position 5 of the indol-3-yl group near the DFG motif (for instance: compound **2v**, log(10^3^/IC_50_) = 6.455). Very close, there is a gray isopleth that is occupied by halogen substituents at position 6 of the same group in several non-active compounds (for instance: compound **2ab**, log(10^3^/IC_50_) = 3.060). Those features together indicate that hydrophobic groups are desired at position 6 of the indol-3-yl group near the DFG motif, but not at position 5. There is other gray isopleth near Asn186 that is occupied by groups at position 6 of the solvent-exposed indol-3-yl or benzofuran-3-yl. The analysis of this isopleth indicates that hydrophobic groups are not desired in this region. For instance, compound **2y** contains a iodide at this position and has log(10^3^/IC_50_) = 3.607; meanwhile, compound **2aa** contains a hydroxyl in this region and has log(10^3^/IC_50_) = 6.022. A very small gray isopleth near Leu188 is occupied by 1-methyl substituents at solvent-exposed indol-3-yl, suggesting that benzofuran-3-yl groups are preferred in this region (instead of the 1-methyl-1*H*-indol-3-yl groups).

Cyan and purple isopleths in Figure 6C are in regions where HB donor groups favor and disfavor the activity, respectively. The cyan isopleth near Asp200 indicates that substituents containing HB donor groups on the indol-3-yl or benzofuran-3-yl near the DFG motif, such as 6-hydroxyl (compound **2x**, log(10^3^/IC_50_) = 5.455), 6-hydroxymethyl (compound **2ah**, log(10^3^/IC_50_) = 6.292), and 7-hydroxymethyl (compound **2av**, log(10^3^/IC_50_) = 5.292), increase the inhibitory activity. Other cyan isopleth is near the backbone carbonyl of the residue Ile62, which is occupied by hydroxymethyl substituents at position 6 of the solvent-exposed benzofuran-3-yl of several active compounds (for instance, compound **2v**, log(10^3^/IC_50_) = 6.596). Finally, a purple isopleth is located near the backbone carbonyl of the residue Pro136, suggesting that HB donor groups are not desired in this zone. In fact, compounds containing a 1-methyl-indol-3-yl have a better inhibitory activity than [36] **2f** and **2i** (log(10^3^/IC_50_) = 3.133 and 3.444, respectively).

Given the model CoMSIA-NCM-SHD reported here, it is easier to explain the trend of the potency of the NCMs against GSK3β. This information can orient in chemical synthesis of new candidates. Some maleimide derivatives studied in this work were previously studied by Fang et al [36]. These authors extracted the compounds (only NCMs) from the reference [21] and constructed CoMFA and CoMSIA models using 30 compounds in the training set and 8 compounds in the test set. They did not determine the active poses (using docking) before QSAR calculations; therefore, their approach only considers ligand-based alignment. The goal of the work of Fang et al. was the construction of predictive models (of diverse GSK3β inhibitors) for identifying new hits with topologically diverse scaffolds. Instead, our approach has the purpose of reporting the structural characteristics of the active conformations of the maleimide derivatives (NCMs and MCMs) inside GSK3β, and developing CoMSIA models (with atom fit alignment rules based on receptor-ligand bound conformations) that describe the structure-activity relationship.

## Conclusions

The structure–activity relationship of maleimides derivates as GSK3β inhibitors was studied by using docking and QSAR methods. Some characteristics of these compounds that explain their activities were described such as their orientation and the interactions that they establish with the residues located in the binding site. The most interesting finding is that the most active maleimides adopt a propeller-like conformation. A CoMSIA model was derived including steric, hydrophobic, and HB donor fields. A map of the characteristics that are desired for highly active compounds, and their relation with the residues of the active site, are presented.
